# A neuro‐behavioural model of neophobia

**DOI:** 10.1002/brv.70151

**Published:** 2026-02-24

**Authors:** Arik Dorfman, Aziz Subach, Inon Scharf

**Affiliations:** ^1^ School of Zoology, George S. Wise Faculty of Life Sciences Tel Aviv University Tel Aviv 69978 Israel

**Keywords:** behavioural ecology, fear‐generalization, fear‐learning, model, neurology, predation, PTSD

## Abstract

Fear can be defined as the internal neurological state that releases a repertoire of behaviours an animal performs to reduce the effect of an aversive factor. Neophobia, the fear of novelty, is a fundamental behavioural trait observed across a wide range of species from arthropods to humans. It has been extensively studied by ecologists and psychologists for decades. However, despite its roots in fear, there is a surprising lack of connection between the literature on neophobia and the rich body of research on the neurology of fear. Fear has been studied at the hormonal, network, and cellular levels in the context of fear learning, extinction, and innate responses. Herein, we aim to link the phenotypic expression of the fear of novelty with its underlying neurological mechanisms. Using the mammalian brain as a model system, we construct a neurobiologically based conceptual model to explain the factors influencing neophobic behaviour. We then review existing studies through the lens of our model and demonstrate its utility in exploring natural selection and phenotypic plasticity by integrating behavioural experiments and neurological insights. While behavioural ecology has traditionally focused on observable behaviour without considering the neural mechanisms behind it, we argue that incorporating neurological knowledge can greatly enhance ecological understanding. In the case of neophobia, such integration is not only possible but necessary, and thus the connection between behaviour and brain is both timely and essential.

## I. INTRODUCTION

A purely mechanistic definition of fear can be an internal, neurological state that generates the sum of actions an animal performs to reduce the effect of an aversive factor (Adolphs, 2013). Being reluctant to eat bitter food or even hiding in a shelter when it is too hot is considered a manifestation of fear. This may be somewhat different from the lay person's definition of fear, but it is useful because it allows comparison of fear reactions across a variety of taxa, even those with the most rudimentary brains (Adolphs, 2013; Pribadi & Chalasani, 2022). While aversion – the avoidance of a certain stimulus – can be triggered by other states than fear, like a stronger motivation for another task than the one studied, for example, we use this term here to reference the aversion triggered by fear alone (Kirkden & Pajor, 2006). Thus, we use the two terms interchangeably, as aversion is a behavioural manifestation of fear and is often used as an indicator of fear (Pribadi & Chalasani, 2022).

Fear is sometimes triggered by stimuli only because they are new to the organism. This type of fear is termed neophobia (Greenberg & Mettke‐Hofmann, 2001; Hughes, 2007). Neophobia, or the fear of the new, has been documented across many taxonomic groups, including insects, reptiles, fish, and humans (Pliner, 1994; Cohen, Benjamini & Golani, 2015; Brown *et al*., 2018; Szabo & Ringler, 2023). Its expression correlates with the species' ecology: species differ in their neophobia level according to their habitat, diet, and trophic level (Greenberg, 1990; Greenberg & Mettke‐Hofmann, 2001; Mettke‐Hofmann, Winkler & Leisler, 2002). Neophobia is also considered a personality trait – some individuals within the same population are consistently more neophobic than others, typically characterizing ‘shy’ individuals (Rasolofoniaina, Kappeler & Fichtel, 2021; Takola *et al*., 2021; de Meester, Pafilis & van Damme, 2022; Miller, Garcia‐Pelegrin & Danby, 2022; Ventricelli *et al*., 2022). Despite the extensive body of literature on the topic, there is no consensus on how neophobia should be defined (Greenberg & Mettke‐Hofmann, 2001; Réale *et al*., 2007). For example, some define it simply as the ‘fear of new situations’ (Beissinger *et al*., 1994, p. 54), while others describe it as ‘…avoidance of an object or other aspect of the environment solely because it has never been experienced and is dissimilar from what has been experienced in the individual's past’ (Greenberg & Mettke‐Hofmann, 2001, p. 125). Here, we adopt the broad definition of neophobia as an aversive response to novelty. But what constitutes an aversive response? And what exactly is novelty?

Although aversion can be measured experimentally, there is no consensus on how it should be measured, even within the same discipline. In many studies, it is assessed by recording the latency to approach a novel object or a new area within an experimental setting (Galef Jr., 1970; Cowan & Barnett, 1975; Desforges & Wood‐Gush, 1975; Middelkoop, Kemp & Bolhuis, 2020). In early ethological and psychological research, an aversive response was often defined as the avoidance of a novel section of a maze or a cage when the animal was given a choice (Corey, 1978; reviewed in Hughes, 2007). Typically, a previously blocked area was made accessible, and the subject was released into the experimental arena. If the animal entered the familiar section, it was deemed neophobic; if it explored the new section, it was considered neophiliac (attracted towards novelty). Another common proxy for neophobia involves placing a novel object next to a familiar food tray or replacing the food tray with a new one (Barnett, 1958; Coleman & Mellgren, 1994; Ensminger & Westneat, 2012; Crane & Ferrari, 2017; Suzuki *et al*., 2021). The longer it takes the animal to approach the food, the more neophobic it is presumed to be. Although an increasing number of recent studies are conducted in more natural settings or semi‐natural enclosures (Webster & Lefebvre, 2001; Greggor *et al*., 2016; Brown *et al*., 2018; Rasolofoniaina *et al*., 2021), these measures are still the main proxies for aversion (Kimball & Lattin, 2024). Whether these different proxies are comparable remains an open question (Kimball & Lattin, 2024). For instance, measuring neophobia based on latency to explore a new environment may be influenced by the animal's motivation to explore, not just its level of fear (Mettke‐Hofmann *et al*., 2006). The answer to the question of what aversion is remains, therefore, controversial.

An even more elusive question is what novelty is. Most studies simply use an object, food item, smell, or environment that the animal has never previously encountered (Mettke‐Hofmann *et al*., 2006; Kelly *et al*., 2022; Miller *et al*., 2022). While this definition is logical, it is not without problems. For example, you may never have encountered a three‐headed dragon, but your hesitation to approach one would likely reflect more than mere aversion to novelty. In other words, some stimuli are inherently fear‐inducing for certain species. To account for this, researchers have distinguished between ‘evolutionarily novel’ and ‘evolutionarily familiar’ stimuli (Saul and Jeschke, 2015; Crane *et al*., 2020). Evolutionarily familiar stimuli (such as large moving things that are perceived as large predators, as in the dragon example) may not have been encountered during an individual's lifetime, but they are still likely to provoke a species‐specific response due to a long history of coevolution with sufficiently similar threats.

Herein, we extend this idea further and argue that no stimulus is perceived as completely new. We support the claim that every new, consciously perceived stimulus is immediately evaluated and placed along a cognitive continuum of experiences, with varying degrees of similarity to past experiences (Peer *et al*., 2021). Specifically, we propose that fear can be considered as one axis of this similarity, and that neophobia represents a combination of learned fear generalization, extinction processes, and innate fear responses.

Fear generalization is a cognitive process in which an animal that has previously encountered an aversive stimulus will subsequently treat any related stimuli as aversive (Dunsmoor & Paz, 2015). For example, if an individual was once poisoned by food A, they may later fear and avoid food B, provided that food B somewhat resembles food A. The neurological correlates of this process have been extensively studied in mammals, primarily rats and humans (Asok, Kandel & Rayman, 2019). These include brain structures involved in memory retrieval, consolidation, and fear reactions, such as the hippocampus, frontal cortex, and the amygdala complex (Roozendaal, McEwen & Chattarji, 2009; Dunsmoor & Paz, 2015; Asok *et al*., 2019). By contrast, innate fears act as cognitive ‘shortcuts’ that trigger rapid defensive responses to a stimulus perceived innately as frightening, such as heights or looming shadows, without prior learning (Ren & Tao, 2020).

Our goal here is to relate the phenotype, the aversive response to novelty, to its mechanism, the underlying anatomy of the brain, by using the mammalian brain as a model system. Using this model, we suggest a conceptual model that breaks down the determinants of neophobia into four components. We then provide examples based on previous research on how to use an experimental approach to pinpoint which factors are responsible for differences in neophobia across species and populations. In the following sections, we outline current challenges in understanding neophobia. We briefly review the mechanisms of fear generalization, extinction, and innate fear. We then propose how an aversive reaction to novelty can be interpreted as a manifestation of these neural processes. Finally, we discuss how future behavioural studies on neophobia can be analysed in light of this hypothesis and suggest potential ecological and evolutionary implications based on these considerations.

## II. PROBLEMS OF DEFINITION AND PREDICTION

Neophobia is used as an umbrella term encompassing a range of phenomena, including both innate and learned fear responses and exploratory impulses (Bolbroe, Jeppesen & Leirs, 2000; Mettke‐Hofmann *et al*., 2006; Cohen *et al*., 2015). Consequently, several attempts have been made to subcategorize it into types of neophobia. To reduce confusion, Crane *et al*. (2020) defined five major types of neophobia: gustatory, predator, social, object, and spatial neophobia, based on a broad review of the literature. While these categories may offer a convenient way to organize findings, their explanatory power seems limited. For one, these types often overlap. For example, does a scarecrow elicit object or predator neophobia in ravens? On the one hand, it is an inanimate object, suggesting object neophobia; on the other hand, ravens may perceive it as a predator, implying predator neophobia. Since we do not know how a raven perceives this stimulus, we cannot decisively assign it to one category. Similarly, spatial neophobia may stem from fear of unknown predators (predator neophobia) or unfamiliar food sources (gustatory neophobia) in the new environment, making clear classification difficult.

A second potential problem with this classification is that it ignores the degree of novelty of the presented stimulus. To address this, Crane *et al*. (2020) discussed another distinction (after Saul and Jeschke, 2015): between ‘evolutionarily novel’ (or induced) neophobia and ‘evolutionarily familiar’ (or baseline) neophobia. Induced neophobia is a reaction to stimuli that did not coevolve with the species, while baseline neophobia involves a reaction to stimuli with which the species shares an evolutionary history (Crane *et al*., 2020). This classification attempts to distinguish between innate responses to new, but evolutionarily familiar, threats (e.g. a predator taxidermy) and entirely novel ones (e.g. an amorphous sculpture).

**Fig. 1:**
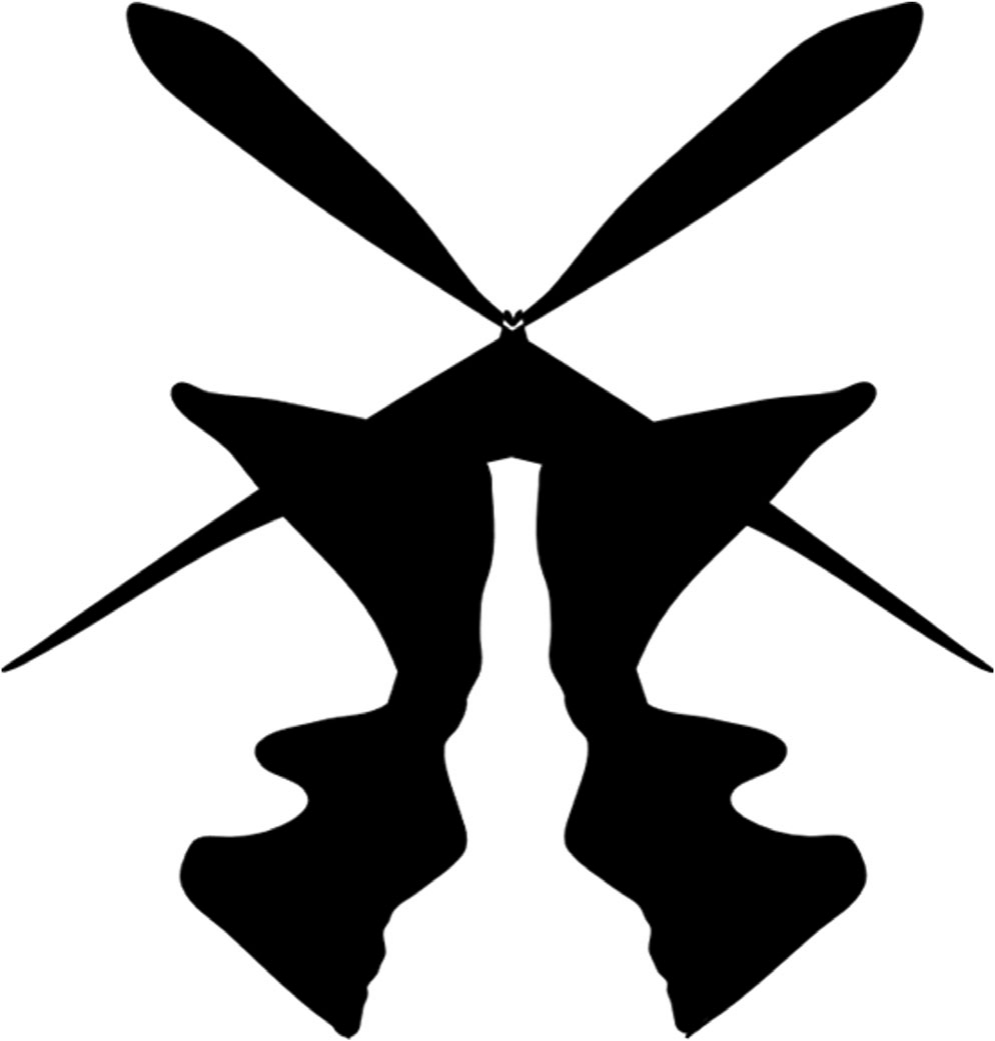
An example of a Rorschach test image. Although the image is a random sketch, it is not perceived as entirely novel: it may remind the observer of two human faces, a moth, a bottle, or other shapes. This simple example illustrates how the brain links each stimulus, no matter how unfamiliar, to past experiences using a cognitive map. In doing so, it rapidly evaluates novel situations, such that no experience is entirely new or neutral.

However, this division rests on a problematic assumption – that in cases of evolutionarily novel neophobia, the animal perceives the stimulus as completely novel. We claim that this is never the case. An intuitive analogy is the Rorschach test (Fig. 1), in which subjects are shown abstract drawings and are asked what they see. Although the images are unfamiliar and amorphic, subjects still interpret them through the lens of prior experience (Wood *et al*., 2000; Gronnerod, 2004; Aschieri & Pascarella, 2021). This illustrates well a key notion in neurology: each experience is a point on a cognitive landscape of relationships, and new experiences are immediately evaluated according to their likeness to prior ones (Behrens *et al*., 2018). Accordingly, even in cases of evolutionarily novel neophobia, the stimulus is assessed as more or less similar to past experiences. Therefore, the evolutionarily novel/familiar dichotomy is incomplete, as it fails to capture the degree of novelty of the experimental stimulus explained by the animal. As argued above, a stimulus cannot be perceived as entirely new, only more similar or less similar to past experiences.

In our view, the only meaningful way to define neophobia and resolve the confusion that may be caused by artificial definitions is to anchor the definition of neophobia in the anatomical structures that generate it. That is, neophobia should be studied not only as a visible behavioural phenomenon by the experimenter but also through the lens of neural processes in the brain. In the following sections, we propose a neuro‐behavioural model of neophobia that aims to explain how neophobia works by considering the underlying anatomical basis of stimulus evaluation and fear generalization processes in the mammalian brain, as a model system.

One important purpose of this neurobiological knowledge for ecologists and ethologists, in our opinion, is to generate grounded hypotheses and connect the evolution of behaviour to the evolution of neurological pathways, assuming that the general and basic mechanisms of learning and innate knowledge are conserved. Thus, we discuss the mammalian brain mainly because it is well studied. Describing this system in depth can help us understand the general mechanisms behind the aversive reactions of other taxa to novelty. We believe that our discussion can be broadened to other taxa, because aversion to threatening stimuli and learning to avoid them are probably universal (Stankowich & Blumstein, 2005; Pribadi & Chalasani, 2022). Even animals without a well‐defined brain, like insects, are capable of fear learning (Giurfa, 2015; Kinoshita & Homberg, 2017). Additionally, there are analogous brain parts to the amygdala in Aves (Yamamoto *et al*., 2005), and virtually all amniotes have a rudimentary hippocampus (Striedter, 2016).

For this reason, we first provide an overview of the role of the relevant brain structures in mammals and then use it to generalize the physiological mechanisms behind neophobia.

## III. THE COGNITIVE MAP AND FEAR PROCESSING IN MAMMALS

We propose that every new, consciously perceived stimulus is evaluated against previous experiences or innate fears before the animal decides how to respond. To compare incoming stimuli to past experiences, animals must retain memories of past experiences.

In the mammalian brain, neuronal pathways run from the primary sensory areas, through the association cortices, to the hippocampus and associated structures (including the parahippocampal and perirhinal cortices, entorhinal cortex, dentate gyrus, and subiculum), and then back to the association cortices (Kandel *et al*., 2012). Each of these regions plays a distinct role in memory formation and retrieval. The sensory areas are where the sensory inputs are first processed. In the association cortices, sensory information from multiple sensory modalities is integrated. This integrated input is then processed in the hippocampus, where it can be stored as long‐term memory. As part of this integration, memories are assigned an ‘emotional charge’ by the amygdala (Davis, 1992; Elzinga & Bremner, 2002), a structure primarily associated with fear and fear learning. It also takes part in the storage of the emotional components of long‐term memories (Kandel *et al*., 2012). The hippocampus plays a central role in memory consolidation (i.e. converting short‐term memories into long‐term ones) and is also important for memory retrieval and context mapping (Scoville & Milner, 1957; Eichenbaum *et al*., 1999; Kandel *et al*., 2012; Maren, Phan & Liberzon, 2013).

This general framework outlines the neural pathway involved in the consolidation of explicit memories – that is, memories of facts and personal experiences (Schacter, 1987). We next focus on the specific roles of the hippocampus (and related structures) and the amygdala within this pathway.

### (1) The cognitive map

Apart from its role in memory, the hippocampus is often described as the structure that harbours the ‘cognitive map’. This term was first coined by Tolman (1948) (see Peer *et al*., 2021), based on a series of experiments with laboratory rats. Tolman (1948) concluded that the brain must contain a map‐like neuronal representation of the world, in which coordinates are assigned to each stimulus to encode its relationship with others. In Tolman's view, the map should be multimodal, integrating information from various ‘stimulus spaces’, such as physical space, and object size and colour. Subsequently, O'Keefe & Nadel (1978) discovered compelling neuronal correlates of this spatial cognitive map in the form of ‘place cells’ in the hippocampus. These are neurons that fire when an animal occupies a specific location in space. Together, they can encode an animal's entire spatial environment, effectively forming a spatial cognitive map of its world. Subsequently, additional cell types were later identified in the hippocampus and related regions that encode further aspects required of spatial navigation, such as the direction of the animal's head, boundaries of the arena, and distance from other individuals (Taube, Muller & Ranck, 1990; Hafting *et al*., 2005; Solstad *et al*., 2008; Krupic, Burgess & O'Keefe, 2012; Sarel *et al*., 2017; Gauthier & Tank, 2018; Omer *et al*., 2018; Høydal *et al*., 2019).

More recently, accumulating evidence suggests that the cognitive map is not limited to encoding spatial information but also encodes all types of goal‐related stimuli in a map‐ or graph‐like structure (Behrens *et al*., 2018; Peer *et al*., 2021). For example, navigating through auditory frequencies that are associated with rewards or social hierarchies in competitive tasks (e.g. games) elicits neural activity in the hippocampus and entorhinal cortex that mirrors patterns observed during spatial navigation through a physical space (Wilson *et al*., 2014; Eichenbaum, 2015; Schuck *et al*., 2016). These findings support the existence of a multimodal cognitive map in the mammalian hippocampus and related structures, as originally envisioned by Tolman (1948).

In the context of fear, the hippocampus works in concert with the amygdala to associate aversive experiences with their surrounding context, linking, for example, a painful experience to a preceding sound (Baldi, Lorenzini & Bucherelli, 2004). By learning which contexts predict aversive events, animals learn what to fear. As with other goal‐relevant stimuli, relationships between threatening and safe contexts are likely organized in a map‐ or graph‐like representation within the hippocampus (Peer *et al*., 2021). This enables animals to classify a new context as threatening or safe based on past experience.

Both roles of the hippocampus, its involvement in memory formation and its capacity to encode structured relationships among stimuli, are important for determining how an animal responds to a novel stimulus, i.e. whether to behave defensively or not. However, the way an animal decides to what degree a stimulus should be similar to a familiar aversive one in order to act defensively depends on fear generalization, extinction, and innate fear mechanisms.

### (2) Innate fears

Some stimuli evoke fear even in newborn animals – these are termed innate fears (Ren & Tao, 2020). Innate fears are species specific: for example, rat cubs fear fox urine, and humans readily learn to fear snakes (Öhman & Mineka, 2003; Wernecke *et al*., 2015). This is ecologically logical – when an animal has no prior information about the world, it is safer to be vigilant towards all long, slithering creatures than to fear only a specific, venomous snake species, for example (Öhman & Mineka, 2003). Of course, other stimuli also elicit innate fear, such as heights, moving objects, looming shadows, and spiders (Ren & Tao, 2020). These fears are imprecise and do not initially involve cortical processing in infants (Chan *et al*., 2011). Instead, they often trigger rapid responses *via* direct pathways from the sensory cortices directly to the amygdala, and from there to the thalamus and motor areas, generating a rapid behavioural response, bypassing the frontal cortex (Ren & Tao, 2020).

As the animal matures and gains experience, innate fears become more refined and specific. This occurs through the process of fear extinction, a process in which the animal actively learns to suppress fear responses to certain stimuli (Hernandez *et al*., 2022). Fear extinction is considered to be a form of active learning rather than forgetting, while both memories coexist in the brain and compete for downstream effects and behavioural expression (Quirk, 2002). The reappearance of fear after extinction training supports this view (Herry & Garcia, 2002). Thus, with experience, animals actively learn which innate fears are truly worth fearing. Beyond these innate fears, however, animals must also acquire new fears, which they cannot be born with (fear of traffic wardens, for example) – this is usually regarded as a type of associative learning, that is, in our discussion, connecting an unrelated signal to an aversive experience (Pearce & Bouton, 2001). In mammals, this is an important function of the amygdala–hippocampus axis.

### (3) Fear learning and generalization

When new memories are formed, the amygdala plays a key role in learning which new stimuli are worth fearing and which are harmless. It does so by associating an aversive experience, processed by the brain's sensory areas (e.g. a neuronal representation of pain or stress), with its predictor, represented in the hippocampus (e.g. a memory or a neuronal representation of a room where an electric shock was previously received) (Nachman & Ashe, 1974; Davis, 1992; LeDoux, 2003; Gale *et al*., 2004). The amygdala is also involved in the retrieval, not just the formation, of emotionally charged memories through its connection with the hippocampus (Izquierdo & Medina, 1993; Izquierdo *et al*., 1997; Kandel *et al*., 2012). As an animal encounters a known ominous setting, the amygdala, which learned to connect the setting with a negative experience, by the neurological input from the hippocampus that represents the structure of the context, integrated with neurological input from sensory cortices that signal pain, raises an alarm by activating motor areas of the brain (Chaaya, Battle & Johnson, 2018).

Evidence for this role comes from various studies, such as those including lesions, neuronal inhibitors, and hormones. For example, animals with amygdala lesions fail to associate a stimulus with an experimentally induced pain or show impaired learning (Nachman & Ashe, 1974; Scott *et al*., 1997; Anderson & Phelps, 2002; Reilly & Bornovalova, 2005). Moreover, stress hormones, such as noradrenaline and cortisol, are known to modulate the strength of aversive memory formation through their specific effect on the amygdala (Ferry, Roozendaal & McGaugh, 1999; Roozendaal, Barsegan & Lee, 2007; Roozendaal *et al*., 2009).

Because no two stimuli are exactly alike, it is not adaptive to react defensively only to the precise stimulus that caused harm in the past: after being stung by a scorpion, one would not fearlessly pet a slightly smaller one, for example. Animals benefit from generalizing negative experiences to similar cues, enhancing future threat avoidance. This process, known as fear generalization, has become a prominent research topic, especially in mammals (Asok *et al*., 2019; Dunsmoor & Paz, 2015). It is an active, carefully controlled, energy‐intensive process, and it is governed at the circuit, micro‐circuit, and cellular levels (Davis & Zhong, 2017; Asok *et al*., 2019). Problems with this process are linked to post‐traumatic stress disorder (PTSD) in both humans and animals (Elzinga & Bremner, 2002; Cohen *et al*., 2023), where individuals over‐generalize traumatic events and react fearfully in harmless situations. This process mainly involves pathways between the amygdala and related sensory cortices (Morris, Buchel & Dolan, 2001; Elzinga & Bremner, 2002). Thus, generalizing the right amount from past fearful experiences, triggering caution in future novel situations, is a vital mechanism for survival.

To summarize briefly the anatomy of the perception and memorization of a new experience: a stimulus is detected through the sensory organs, and the information is transmitted to the hippocampus (Kandel *et al*., 2012). There, the stimulus is evaluated and mapped relative to previous experiences on the animals' cognitive map (Peer *et al*., 2021). During the overall evaluation of the experience, the amygdala assesses the risk or fear‐related aspects of the experience through its interactions with the hippocampus and sensory cortices (LeDoux, 2003). A behavioural response is chosen and executed. The experience is then encoded in both cortical and limbic regions as a consolidated memory and may undergo generalization over time (Asok *et al*., 2019). If the experience was aversive, a future stimulus that merely resembles the original, even without involving direct aversive input (such as pain), can provoke a defensive reaction, as the amygdala–hippocampus axis categorizes it as threatening based on the generalization of prior experience (Chaaya *et al*., 2018).

In the following sections, we use this framework to build a conceptual model that may help behavioural ecologists interpret the results of neophobia‐related studies better. But first, we would like to expand the discussion to invertebrates for the sake of generalizing our scheme.

## IV. A NOTE ON INVERTEBRATES

This review mainly discusses the mammalian brain as a model; however, our conclusions can be applied to other taxa with a very different neuronal system organization: invertebrates. Invertebrates, such as gastropods, insects, and roundworms, are capable of associative fear learning, that is, they are capable of learning to avoid neutral stimuli (Pribadi & Chalasani, 2022). Furthermore, they can integrate stimuli from different sensory modalities during the learning process (Pribadi & Chalasani, 2022; Thiagarajan & Sachse, 2022). This fact holds despite the strikingly different organization of invertebrate brains in comparison to mammals. Insects have a decentralized nervous system. That is, most of their movements and immediate responses bypass processing in the higher‐order centres and rely solely on the nerve cord (Wessnitzer & Webb, 2006). However, they have neuronal correlates to the mammalian higher‐order processing centres (Thiagarajan & Sachse, 2022). For example, mushroom bodies are brain structures that play a major role in the integration of stimuli and learning (Heisenberg, 2003; Wessnitzer & Webb, 2006). Mushroom bodies also play a role in context generalization of visual cues, a similar role to that of the hippocampus (Liu *et al*., 1999; Zars, 2000). A good example of the analogous function of the insect's brain to the mammalian brain in terms of fear learning is provided by Liu *et al*. (1999), who trained fruit flies (*Drosophila melanogaster*) to associate a specific background pattern with aversive high temperatures and then switched the background colour. Flies with ablated mushroom bodies did not avoid the same pattern (but a different colour), contrary to intact flies. This finding illustrates the fact that insects can learn to avoid a novel stimulus based on the generalization of a known, aversive one, and that the mushroom bodies are responsible for this ability.

**Fig. 2:**
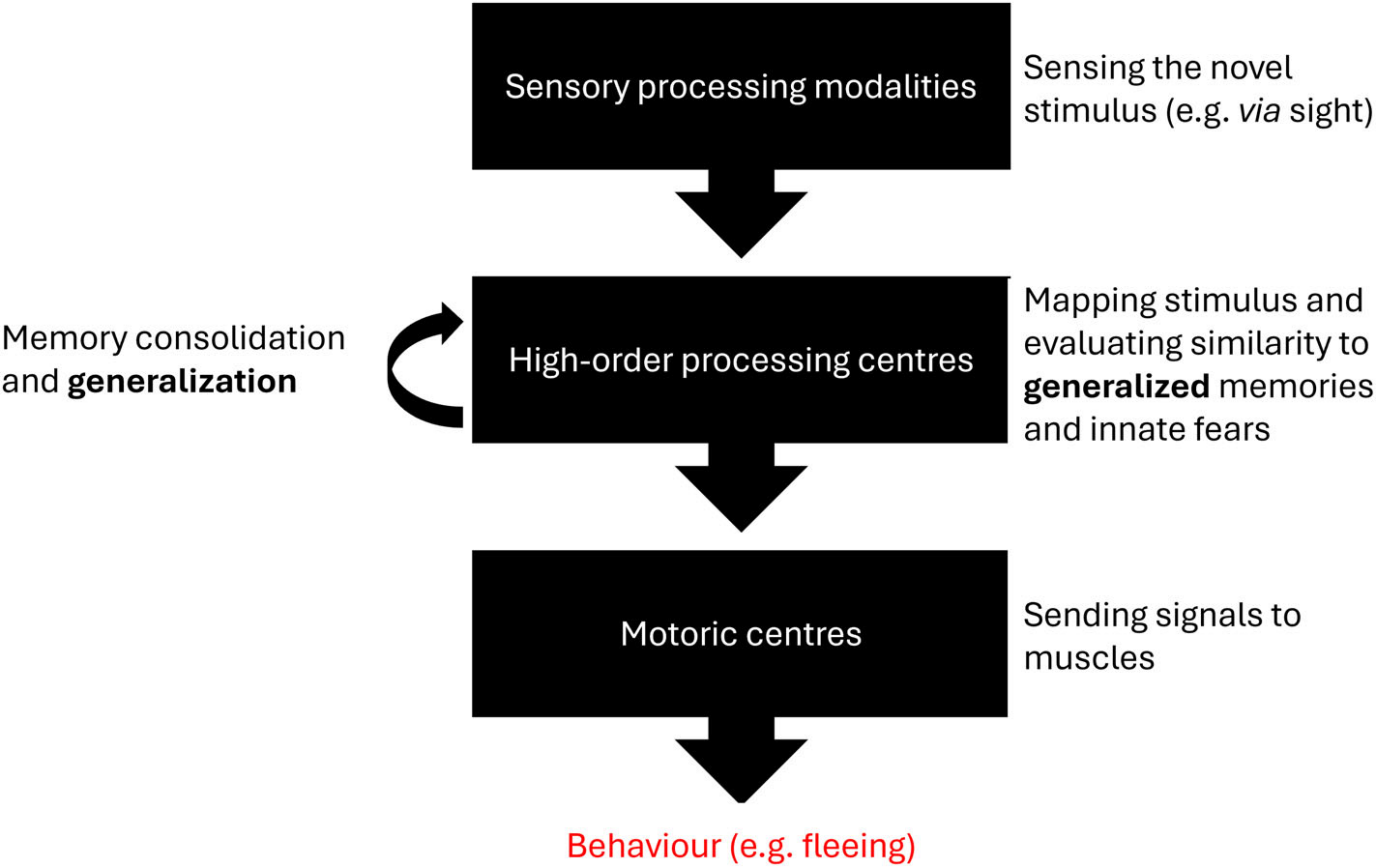
A generalized pathway of assessing and acting upon a novel stimulus. A stimulus enters the nervous system through the sensory organs, is integrated, and sent to the higher‐order processing centres. There, it is evaluated as similar/different from past experiences, and its memory is consolidated and generalized. Based on this evaluation, the motoric centres send signals to the muscles, and the behaviour is released. This scheme is general, and may therefore apply to a variety of taxa, from mammals to insects. Of course, many details of intricate feedback loops and specific brain structures are missing in this representation for simplicity.

Thus, even insects are capable of some generalization of aversion and assess the similarity of novel stimuli to generalized memories, as discussed in Section III for mammals, and some roles of the neural structures responsible for this ability are known. Fig. 2 presents a scheme for the generalized pathway for assessing novel stimuli.

## V. A CONCEPTUAL NEURO‐BEHAVIOURAL MODEL FOR NEOPHOBIA

Our model builds mainly on the role of the hippocampus as a multimodal cognitive map, where consciously perceived stimuli are organized (along one of many axes) by their similarity to known aversive stimuli. In the following text, we refer to the number of stimuli sufficiently similar to past aversive experiences to elicit fear as ‘fields of aversive stimuli’.

**Fig. 3:**
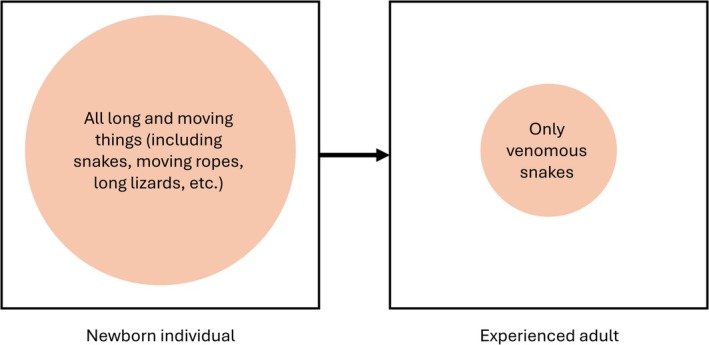
Example of a field of stimuli (orange circles) that elicit a fear response when encountered by an individual. As the individual ages, innate fears become more specific through fear extinction, resulting in a smaller set of stimuli triggering fear compared to the broader set defined by innate fears.

Animals are born with innate fears; that is, there are novel stimuli that can evoke fear upon first encounter. For example, a newborn exposed to a slithering string may react fearfully, despite never having seen anything like it before, due to an innate fear likely shaped by coevolution with snakes (Öhman & Mineka, 2003). At this stage, the animal possesses broad fields of aversive stimuli that can trigger neophobic reactions due to innate fears – such as long, moving objects (Fig. 3). With experience, fear extinction processes gradually narrow these fields, as the animal learns to ignore some innate fears that prove harmless or are unaccompanied by pain or stress (Furini, Myskiw & Izquierdo, 2014).

The animal also gradually learns new fears through the learning processes discussed in the previous sections. It generalizes these fears; that is, the number and size of the fields of stimuli that elicit fear increase with fear learning and generalization (Asok *et al*., 2019). As the animal accumulates negative experiences, more novel stimuli that are sufficiently similar to previously aversive ones will trigger neophobia. Moreover, the intensity of generalization influences the size of the fields – the greater the generalization, the more novel stimuli will evoke a fear response.

The intensity of generalization depends on several factors. More severe and negative past experiences tend to cause stronger generalization. Consider, for example, PTSD, which affects humans and probably also wild animals (Cohen *et al*., 2023). In PTSD, a very traumatic experience can cause fear long after the experience itself, as well as fear of otherwise benign stimuli (Elzinga & Bremner, 2002). Similarly, early‐life aversive experiences are especially impactful and often lead to stronger generalization (Elliott & Richardson, 2019). Fear generalization intensity also depends on species or sex, with some individuals being more prone to generalizing fear than others (Keiser *et al*., 2017).

**Fig. 4:**
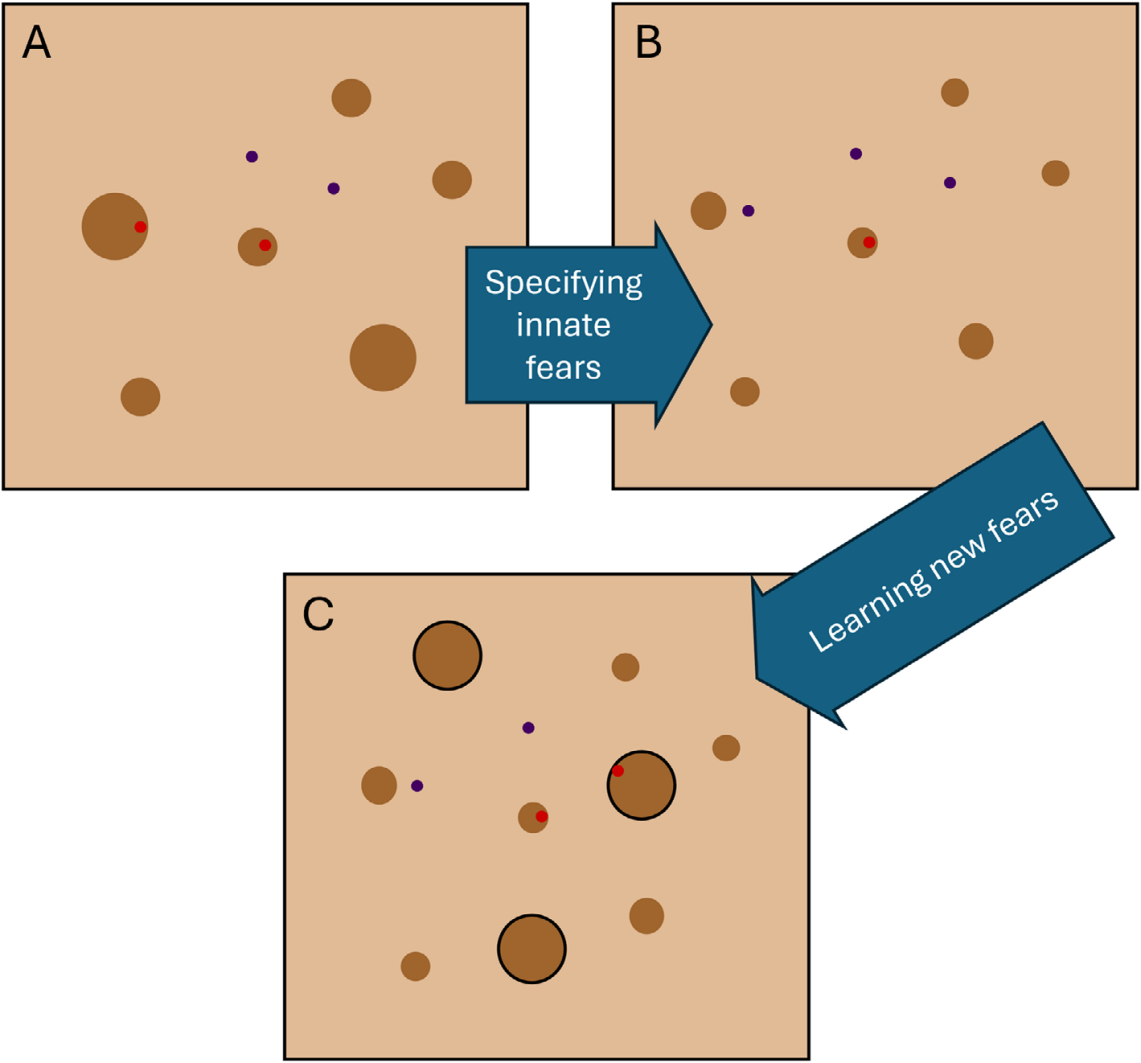
The ‘raindrop model’: brown circles = fields of fear‐eliciting stimuli; red dots = novel stimuli that would elicit neophobia; purple dots = novel stimuli that do not elicit neophobia; light brown background = the ‘fear space’ within the cognitive map. (A) Animals are born with innate fears, which initially span a broad field of stimuli due to their imprecision (large brown circles). (B) With experience, these fears become more specific and accurate, causing the brown circles to shrink in size. (C) Over time, animals learn additional fear‐inducing stimuli and generalize fear to related stimuli (expanding some brown circles again). As animals encounter novel stimuli, their responses depend on how those stimuli are classified: if the stimulus falls within a fear‐associated field (red dots), a fear response is triggered. If it falls outside these fields (purple dots), the animal reacts calmly. Note that this visualization represents a snapshot in time. Like sand that dries out, and new raindrops fall upon it, so fears are extinguished or become more generalized over time.

To visualize these processes, we can use a metaphor: consider raindrop patterns in the sand (Fig. 4). The sand represents the cognitive map, while the raindrops symbolize fields of stimuli that elicit fear responses. Each animal is born with a unique pattern – its set of innate fears (Fig. 4A). Over time, these fields typically shrink due to the extinction of fear (Fig. 4B), while new fields ‘drop’ onto the sand (Fig. 4C), representing learned fears. The size of the field of each drop depends on the factors mentioned above, namely the intensity of the aversive experience and the animal's intrinsic characteristics (its sex, age, learning capacity, etc.). This metaphor represents a snapshot of an animal's ‘fear map’ at a given time, but, as pointed out above, it is ever‐changing. Some learned fears can be easily extinguished, but some (more traumatic or early experienced) are more stable (Elzinga & Bremner, 2002; Elliott & Richardson, 2019). These factors can be grouped into four principal ones, based on the mental determinants of neophobia: (*i*) the number and intensity of past negative experiences; (*ii*) the intrinsic learning capacities and generalization capacities of animals, that is, the innate ability to learn and generalize new experiences; (*iii*) the number and intensity of innate fears; and (*iv*) positive experience with fear‐eliciting stimuli (Table 1).

**Table 1:** The four factors that may influence neophobic reactions and their definitions.

Factor	Innate or learned fear	Definition
(1) Number and intensity of past negative experiences	Learned	As the animal accumulates negative experiences, it increasingly generalizes unfamiliar stimuli as threatening, thereby expanding the range of novel stimuli that elicit neophobia. The more negative the past experiences, the stronger this generalization becomes (Elzinga & Bremner, 2002). Consequently, neophobia will be elicited by a broader array of novel stimuli.
(2) Intrinsic learning and generalization capacities of animals	Innate	The innate ability to generalize new fears depends on various innate factors, including species, personality, and sex (Asok *et al*., 2019).
(3) Number and intensity of innate fears	Innate	Different species possess distinct sets of innate fears, leading to variance in which novel stimuli elicit neophobia (Ren & Tao, 2020).
(4) Positive experience with fear‐eliciting stimuli	Learned	Positive experiences with fear‐eliciting stimuli promote fear extinction (Quirk, 2002). Thus, innate fears become more specific with age, and fewer novel stimuli trigger neophobia.

The model may also be applied to taxa beyond mammals because the mammalian hippocampus shares an evolutionary origin with those of all other amniotes (Striedter, 2016). Although its structure varies among groups, its function appears to be highly conserved. Additionally, both birds and mammals, taxa with relatively large and developed brains, exhibit convergent features in their hippocampi (Striedter, 2016). Birds also possess structural homologs to the mammalian amygdala (Yamamoto *et al*., 2005), and their responses to fear‐inducing stimuli resemble those of mammals in many ways (Papini, Julio & Andrés, 2019). Thus, it is reasonable to assume their categorization of fear is similar and that the model is applicable, at least, to birds.

The model may even be extended to more distantly related taxa. Insects, for example, are capable of both reward‐ and punishment‐based learning, as well as short‐ and long‐term memories, similar to mammals. The brain structures responsible for these are the mushroom bodies (Giurfa, 2015; Kinoshita & Homberg, 2017). While there are no direct analogues to the mammalian hippocampus or amygdala in insect ganglia, insects nonetheless associate natural stimuli with reward or fear in ways that appear functionally similar to mammals. Therefore, it is plausible that the underlying mechanisms of fear classification are comparable. It would be particularly interesting to apply our model in comparative studies of fear classification and learning, using both behavioural assays of neophobia and neurological studies across insects and mammals.

The model's main predictive power, in our view, lies in its division of the determinants of neophobic behaviour into the four distinct factors listed above (Table 1). These factors can be experimentally separated. By using our model, researchers can identify the specific causes or contributing factors behind neophobic behaviour in individual cases. In the next section, we demonstrate how to use this model to interpret findings from previous ecological studies of neophobia, thereby illustrating its predictive power.

## VI. NEW INSIGHTS INTO PREVIOUS RESEARCH

### (1) Neophobia as an adaptive trait

A few studies have shown that domesticated rats and birds are less neophobic than their wild counterparts (Desforges & Wood‐Gush, 1975; Mitchell, 1976; Suzuki *et al*., 2021). This finding makes sense from an ecological perspective: the cost of mistakenly interpreting a harmful novel stimulus as benign is higher in risky environments, whereas in safer habitats like captivity, increased exploration is advantageous (Bouskila & Blumstein, 1992; Johnson *et al*., 2013). But is this an example of natural selection, or merely phenotypic plasticity?

Importantly, both domesticated and wild animals in these studies were reared in captivity in the same conditions, meaning that any observed differences are likely innate. According to factors 1 (number and intensity of past negative experiences) and 4 (positive experience with fear‐eliciting stimuli) of our model (Table 1), we would expect no differences between the groups. Moreover, since the animals were not exposed to aversive experiences, such as predators or rancid food, they had no opportunity for fear learning, making it unlikely that factor 2 (intrinsic learning and generalization capacities of animals) influenced the results.

According to our model, the trait underlying neophobia and subject to selective pressure in this case is factor 3: number and intensity of innate fears. This implies that to study the natural selection of neophobia, researchers should focus on the neural circuits that regulate innate fears and the genes that control them. For example, Jesuthasan & Mathuru (2008) presented an in‐depth summary of studies of the zebrafish (*Danio rerio*) alarm response – a response to the scent of injured skin of conspecifics, including the neurological and genetic correlates of the response. It might be interesting to test the effects of these physiological correlates on the degree of neophobic responses. Additionally, circuits related to innate fears have been studied in detail in rodents. These, too, can serve as a good model for studying the natural selection shaping neophobia (Carli & Farabollini, 2022). While the conclusion that natural selection shapes neophobia *by* shaping the physiological processes that govern the intensity of innate fears follows directly from our model, it has, to the best of our knowledge, been overlooked in the literature.

### (2) Invasion fronts and city dwellers

Many studies have compared the neophobic responses of different populations of the same species, focusing on individuals that live in cities *versus* those in rural areas (Sol *et al*., 2011; Greggor *et al*., 2016; Riyahi *et al*., 2017). Other studies have examined invasive birds at invasion fronts compared to those in original populations (Liebl & Martin, 2012, 2014; Magory Cohen & Dor, 2019). In both cases, animals inhabiting an evolutionarily novel habitat, city or front, are generally less neophobic (but see Miranda *et al*., 2013).

Several explanations are possible. One is natural selection: individuals with fewer innate fears (factor 3) or individuals with reduced generalization of fearful experiences (factor 2) may have a selective advantage in anthropogenic habitats. Supporting this, Møller (2010) found that city birds exhibited less variation in flight‐initiation distance, a proxy for fear of predators, suggesting a selective filter that favours ‘bolder’ individuals in urban settings. Alternatively, reduced neophobia may result from phenotypic plasticity, caused by experiencing fewer or weaker negative events while living in a safer environment (factors 1 and 4).

**Fig. 5:**
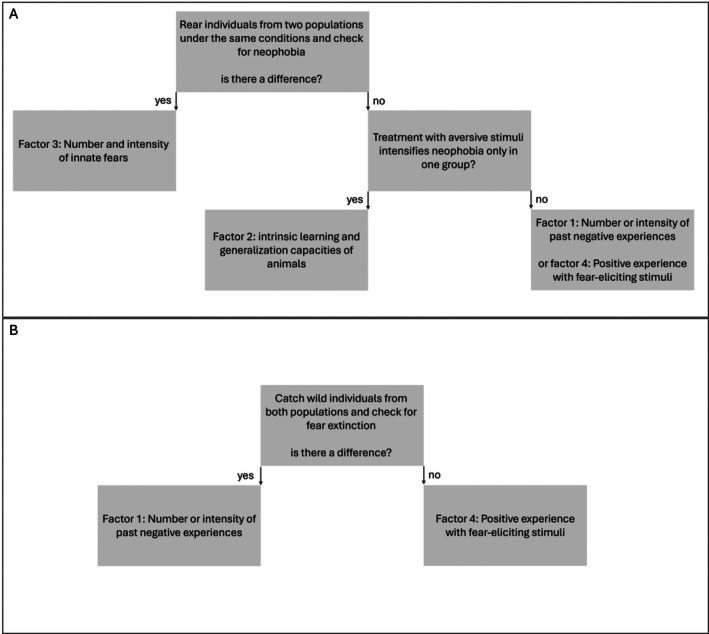
Suggested workflow for identifying the mechanisms underlying differences in neophobia between two populations.

To determine whether these population differences are genetic, controlled laboratory experiments could be conducted in which laboratory‐reared individuals from both groups would be tested for their neophobic responses. If a consistent difference in neophobia between the populations is observed under those conditions, it would suggest a genetic basis, specifically differences in factor 3 (number and intensity of innate fears). If no such difference is found, further experiments should test the other factors. If aversive stimuli increase neophobia similarly in both groups, the difference observed in the wild likely results from factor 1 (number and intensity of past negative experiences), possibly combined with factor 4 (positive experience with fear‐eliciting stimuli). However, if only the rural/native population becomes more neophobic after such treatment, the difference in behaviour may be due to factor 2 (intrinsic learning and generalization capacities of animals). See Fig. 5A for a visual representation of the proposed flowchart. This flowchart can be further refined to differentiate between factors 1 and 4 by designing experiments that manipulate and control different levels of fear extinction in wild‐caught individuals, thereby testing whether fear extinction capacity differs between groups: if the populations differ in their fear extinction, it is because one group experienced more negative experiences (factor 1; as in PTSD, for example: Elzinga & Bremner, 2002), while if there is no difference, probably one of the groups had positive experiences with initially fear‐eliciting stimuli (Fig. 5B). By applying our model to guide such experimental designs, we can investigate whether a key ecological trait, reduced fear of novelty in invasive species, arises from selection on innate brain organization (i.e. innate fear; factor 3), selection on learning capacities (i.e. fear learning capabilities; factor 2), or phenotypic plasticity (i.e. differences in experience that result in different levels of fear generalization; factors 1 and 4).

Furthermore, there are other invasive taxa that are behaviourally distinct from their native populations, such as cane toads (*Bufo marinus*) in Australia (Shine, 2010; Kosmala *et al*., 2017). These can serve as additional model species for studying the adaptation of neophobic behaviour to a novel habitat and will prepare the ground for cross‐taxon comparative studies.

### (3) Effects of habitat risk

Another factor influencing neophobia is the level of predation risk in the habitat. Animals from risky habitats are typically more neophobic (Brown *et al*., 2013, 2014; Meuthen *et al*., 2016; Feyten *et al*., 2022). The adaptive value is clear: for example, fish that were experimentally exposed to predator cues early in life while in captivity showed higher survival rates in the wild, suggesting that neophobia induced by predatory risk is adaptive (Ferrari *et al*., 2015). This experiment supports factor 1 of our model (number and intensity of past negative experiences) as a cause for the variation in neophobia. However, at the species level, differences may be innate. Species with a long evolutionary history of living in dangerous habitats may possess more innate fears or a more sensitive fear‐generalization mechanism. In such cases, a targeted set of experiments (Fig. 5) could help determine whether neophobic tendencies driven by predation risk are shaped by natural selection rather than by phenotypic plasticity and whether the underlying mechanism is enhanced learning capacity or stronger innate fears.

## VII. DISCUSSION

We link here the phenotypic expression of neophobic behaviour to its anatomical basis in the mammalian brain. Building on this connection, we propose a conceptual model aimed at clarifying the causes of neophobia, grounded in current knowledge of neuronal structures, functions, and processes. Our goal is to support the study of the causes of neophobia across species through behavioural research and to encourage clearer and consistent terminology in this field.

In our model, we address only one of the considerations animals make while deciding whether to approach an object, explore a new environment, or flee from another animal – namely, whether the stimulus is perceived as threatening. Other factors are important and may influence experimental outcomes. For example, researchers failed to detect neophobic behaviour in a study on rats in the wild (Modlinska & Stryjek, 2016). This does not necessarily mean that wild rats are less fearful than laboratory rats, but rather that they may have been more strongly motivated by the food reward offered. Motivation, the drive to pursue a specific goal (Mackay, 1959; Wise, 2004), is an important component of behaviour and can override a fear response. Thus, neophobia (the observed behaviour), is a function of both fear and of motivation. The neural circuits governing motivation are well studied in humans and rodents and are mainly concentrated in the dopaminergic pathway of the mesolimbic system (Salamone & Correa, 2024). In our model, we do not account for the role of motivation in neophobia, as we believe it will complicate the interpretation of the model, making it less useful at this stage for experimental application. Instead, we suggest that when comparing neophobic behaviours across groups, researchers should first ensure that their motivation levels are equal (Kimball & Lattin, 2024). A simple control for motivation levels in experimental design can eliminate the need to factor it into fear‐related analyses.

With this consideration in mind, our model can generate a range of predictions, some of which could even appear contradictory. For example, as discussed above, predation risk increases neophobia by affecting the number of learned fears and potentially enhancing innate fears or fear generalization. However, the model predicts no change in neophobic response due to rhythmically fluctuating risk levels, such as those caused by moonlight in nocturnal prey species (Prugh & Golden, 2014). A novel object presented to a nocturnal prey animal during a full moon (a high‐risk period) or a moonless night (a low‐risk period) should elicit the same level of neophobia, assuming no changes have occurred in the animal's innate fears, learning capacity, recent experience, or motivation. Without the framework of our model, such results may be difficult to interpret, given the repeated assertion in the literature that risk enhances neophobia (Brown *et al*., 2013, 2014; Ferrari *et al*., 2015). The model may also guide geneticists in identifying genes regulating neophobic behaviour. For example, natural selection on neophobic behaviour likely targets innate fears. Thus, genes regulating the pathways that underpin these fears (for example, see Matsuo *et al*., 2021) should be studied in the context of evolutionary pressures on neophobia.

To conclude, we hope this paper will help guide future research in behavioural ecology and other fields and assist researchers in revealing the causes of variation in neophobic behaviour across species. By integrating neurological insights into behavioural studies, our model offers a framework for understanding the evolution and plasticity of animal responses to novelty.

## VIII. CONCLUSIONS

(1) We developed a conceptual model linking neophobic behaviour to specific neural processes, drawing primarily on literature concerning the roles of the hippocampus and amygdala in memory consolidation, retrieval, and fear evaluation.
(2) The model explains how innate and learned fears contribute to neophobic responses, using the metaphor of raindrops on sand to illustrate how experience, brain plasticity, and innate tendencies affect fear generalization.
(3) This framework enables researchers to derive evolutionary insights from behavioural data and helps resolve seemingly contradictory findings in the literature.
(4) Although our model centres on fear‐based decision‐making, we highlight the importance of controlling for motivation in experimental designs to isolate the true effects of fear on behaviour.
(5) We intend our model to support interdisciplinary research by bridging neurology and behavioural ecology, thereby advancing our understanding of the evolution and plasticity of neophobia.

## Data Availability

Data sharing not applicable to this article as no datasets were generated or analysed during the current study.
